# Multi-parametric neuroimaging evaluation of cerebrotendinous xanthomatosis and its correlation with neuropsychological presentations

**DOI:** 10.1186/1471-2377-10-59

**Published:** 2010-07-06

**Authors:** Chiung-Chih Chang, Chun-Chung Lui, Jiun-Jie Wang, Shu-Hua Huang, Cheng-Hsien Lu, Ching Chen, Chih-Feng Chen, Min-Chien Tu, Chi-Wei Huang, Wen-Neng Chang

**Affiliations:** 1 Department of Neurology, Chang Gung Memorial Hospital-Kaohsiung Medical Center and Chang Gung University College of Medicine, 123, Ta-Pei Road, Niaosung, Kaohsiung County 833, Taiwan; 2 Department of Radiology, Chang Gung Memorial Hospital-Kaohsiung Medical Center and Chang Gung University College of Medicine, 123, Ta-Pei Road, Niaosung, Kaohsiung County 833, Taiwan; 3 Department of Medical Imaging and Radiological Sciences, Chang Gung University, 259, WenHua t Road, KueiShan, TaoYuan County, 333, Taiwan; 4 Department of Nuclear Medicine, Chang Gung Memorial Hospital-Kaohsiung Medical Center and Chang Gung University College of Medicine, 123, Ta-Pei Road, Niaosung, Kaohsiung County 833, Taiwan; 5 Department of Psychiatry, Chang Gung Memorial Hospital-Kaohsiung Medical Center and Chang Gung University College of Medicine, 123, Ta-Pei Road, Niaosung, Kaohsiung County 833, Taiwan

## Abstract

**Background:**

Cerebrotendinous xanthomatosis (CTX) is a rare genetic disorder. Recent studies show that brain damage in CTX patients extends beyond the abnormalities observed on conventional magnetic resonance imaging (MRI). We studied the MRI and ^99 m^Tc-ethyl cysteinate dimer single photon emission computed tomography (SPECT) findings of CTX patients and made a correlation with the neuropsychological presentations.

**Methods:**

Diffusion tensor imaging (DTI) and 3D T1-weighted images of five CTX patients were compared with 15 age-matched controls. Voxel-based morphometry (VBM) was use to delineate gray matter (GM) and white matter (WM) volume loss. Fractional anisotropy (FA), mean diffusivity (MD), and eigenvalues derived from DTI were used to detect WM changes and correlate with neuropsychological results. SPECT functional studies were used to correlate with GM changes.

**Results:**

Cognitive results showed that aside from moderate mental retardation, the patient group performed worse in all cognitive domains. Despite the extensive GM atrophy pattern, the cerebellum, peri-Sylvian regions and parietal-occipital regions were correlated with SPECT results. WM atrophy located in the peri-dentate and left cerebral peduncle areas corresponded with changes in diffusion measures, while axial and radial diffusivity suggested both demyelinating and axonal changes. Changes in FA and MD were preceded by VBM in the corpus callosum and corona radiata. Cognitive results correlated with FA changes.

**Conclusion:**

In CTX, GM atrophy affected the perfusion patterns. Changes in WM included atrophy, and axonal changes with demyelination. Disconnection of major fiber tracts among different cortical regions may contribute to cognitive impairment.

## Backgroud

Cerebrotendinous xanthomatosis (CTX) is a rare, autosomal recessive, lipid storage disease. Leukoencephalopathy, signal abnormalities of dentate nuclei of the cerebellum, is frequently reported in conventional brain magnetic resonance imaging (MRI) [1]. Recent studies have shown that brain damage in CTX patients extends well beyond the abnormalities observed on conventional neuroimaging studies [1], and that this maybe responsible for the neurologic manifestations.

Voxel-based morphometry (VBM) is an unbiased, whole brain quantitative method that can detect atrophies of gray matter (GM) and white matter (WM) [2]. Diffusion tensor imaging (DTI) can be used to investigate WM tract pathology since it is sensitive to water diffusion [2]. Fractional anisotropy (FA) and mean diffusivity (MD) [3] can be derived from DTI to provide information on micro-structural changes. FA is a reflection of the degree of directionality of cellular structures within the fiber tracts [4,5], while MD represents the magnitude of diffusion and reveals information on tissue integrity [6]. Analyses of axial and radial diffusivities provide potential measures of the mechanisms that underlie diffusion tensor changes. Tract-based spatial statistics (TBSS) focuses on spatial localization of diffusion-related changes using voxel-wise comparison between patients and normal controls [7]. Diffusion MRI provides quantitative measurements compared with conventional MRI in detecting WM pathology and is better suited for a more comprehensive evaluation of diffusion changes in WM [3].

In this study, we chose 3-dimensional T1 MRI, DTI and 99 mTc-ethyl cysteinate dimer (ECD) single photon emission computed tomography (SPECT) to explore the structural and functional changes in five CTX patients. The multi-parametric modalities provided quantitative measurements which could be compared with age-matched controls and provided a better understanding of the regions being affected in CTX as compared with conventional imaging. Correlations between the neuroimaging modalities and with neuropsychological presentations were also analyzed.

## Methods

### Subjects

Five biochemically and genetically confirmed CTX patients (mean age at examination = 41.4 years), belonging to two families, were included in this study and their demographic data, clinical features, brain MRI findings, and data of neuropsychological tests are listed in Table 1. Genetic mutations in Family I (Cases 1 and 2) were compound heterozygous for a transition of C to T at position 1333 (1333C > T) in exon 8 on one allele, and a transition of G to A at the first nucleotide of intron 7 (IVS7 + 1 g > a) on the other allele. The clinical features and genetic study results of Family II (Cases 3, 4, and 5) have been previously reported [8,9]. In addition, 15 sex-and age-matched healthy subjects from the normative database acted as controls for the neuropsychological testing and brain imaging comparison study. The human ethics committee of Chang Gung Memorial Hospital approved the study [IRB98-1803B].

**Table 1:** Demographic and neuropsychological evaluation of the five cerebrotendinous xanthomatosis patients

	Case 1	Case 2	Case 3	Case 4	Case 5
Gender	male	male	female	male	female
Age (years)	29	27	54	49	48
Age at diagnosis (year old)	24	23	37	32	31
Clinical features					
Cataract	+	+	+	+	+
Mental Retard	mild to moderate	mild	moderate	moderate	moderate
Psychiatric diagnosis	Anxiety	none	none	none	Dysthymia
Epilepsy	+	-	-	-	-
Achilles tendon xanthoma	+	+	+	+	+
Ataxia	+	+	+	+	+
MRI follow up numbers (times)	3	3	6	6	6
Image features at diagnosis and follow up					
Dilation of 4^th ^ventricle at diagnosis	+	+	+	+	+
Peri-dentate WM change	+	+	+	+	+
Peri-ventricular WM	+	+	+	+	+
hyper-intensity					
Cerebellar WM changes	+	+	+	+	+
WAIS-R at diagnosis	64/49/55	71/65/67	55/54/49	50/50/45	50/50/45
(VIQ/PIQ/FIQ)					
WAIS-R (VIQ/PIQ/FIQ)	64/49/55	71/65/67	62/57/55	54/57/49	54/57/49
Mini-mental state examination	24	23	7	6	7
Clinical dementia rating	0.5	0.5	2	2	2
CASI (100)	75.7*	73.8 *	19*	24.2*	25.7*
Mental manipulation (10)	2	7	0	1	2
Attention (8)	8	6	5	5	5
Orientation (18)	18	16	4	4	4
Long term Memory (10)	4	4	0	0	0
Short term Memory (12)	12	9.8	1.5	2.7	3.7
Abstract thinking (12)	10	9	2	5	6
Drawing (10)	6	8	5	5	4
Verbal fluency (10)	6	6	1	1	1
Language (10)	9.7	8	0.5	0.5	0

WAIS-R = Wechsler Adult Intelligence Scale-Revised; CASI = Cognitive ability screening instrument; (+) = present; (-) = absent; WM = white matter; MRI = magnetic resonance imaging.

*score < 5 percentile in CASI, numbers in the parenthesis indicate maximal score;

### Cognitive Testing

The Mini-Mental State Examination (MMSE) [10] and Wechsler Adult Intelligence Scale-Revised (WAIS-R) [11] were used to assess general intellectual function. Cognitive severity was evaluated using the Clinical Dementia Rating (CDR) and Cognitive Ability Screening Instrument (CASI) [12].

### MRI Protocols

MRI was performed using a 3.OT scanner (Excite, GE Medical System, Milwaukee, WI) equipped with echo-planar capability. The structural MRI sequences were as follows: 1) eighteen slices of axial fast spin-echo T2-weighted image (T2WI) (4200/102/0/2[TR/TE/TI/NEX]; field of view (FOV), 24 cm; matrix, 320 × 224; and section thickness, 5 mm), and fluid-attenuated inversion recovery (FLAIR) image (8000/100/2000/1[TR/TE/TI/NEX]; FOV, 24 cm; matrix, 320 × 256; and section thickness, 5 mm); 2) T1 inversion recovery prepared three-dimensional spoiled gradient-recalled acquisition in steady state sequence with parameters of TR 8600 ms, Prep time 400 ms, FOV: 240 mm × 240 mm, slice thickness 1 mm; and 3) DTI with gradients applied in 25 non-collinear directions were acquired using the following parameters: TR/TE = 7000 msec/72 msec, FOV: 240 mm × 240 mm, matrix size: 128 × 128, which lead to an in-plane resolution of 1.875 mm. The data were subsequently interpolated to 1 mm × 1 mm. Thirty contiguous slices of thickness 5 mm were obtained without gap and a *b *value of 1000 s/mm^2 ^was used with one b = 0 image.

### Data pre-processing

Processing of the imaging data was performed using Statistical Parametric Mapping 5 software (Wellcome Department of Cognitive Neurology, London, UK) for VBM and the FSL version 4.0 package for TBSS data.

### VBM

The VBM protocol followed the standard procedures [13]. All T1-weighted images were spatially normalized into the standardized Montreal Neurological Institute space using a 12-parameter affine transformation and non-linear normalization. The study-specific template was then smoothened with an 8-mm full-width at half-maximum (FWHM) isotropic Gaussian kernel. The original images were warped to match the customized templates and re-sliced onto a voxel size of 1 × 1 × 1 mm to minimize partial volume effects. The images were segmented into gray, white and CSF compartments, modulated with Jacobian determinants to compensate for volume changes in non-linear spatial normalization, and smoothened with a 10 × 10 × 10 mm FWHM isotropic Gaussian kernel.

The general linear model was used to assess statistical differences in GM and WM among the patients and controls. Global differences in voxel intensities were used as confounding covariates in an analysis of covariance. Age and gender were considered covariates of no interest to exclude their possible effects on regional GM or WM [13]. An unpaired t-test design in the framework of the general linear model was used for analysis with a significance threshold set at *p *< 0.05 corrected for multiple comparisons across the whole brain [family-wise error (FWE)].

### TBSS of FA, MD and three eigenvalues

Eddy-current distortions and motion artifacts in the DTI dataset were corrected by applying affine alignment of each DW image to the b = 0 image, using functional MR imaging of the brain (FMRIB) Diffusion Toolbox (FSL, version 4.0; http://www.fmrib.ox.ac.uk/fsl) [14]. Voxel-wise statistical analysis of the FA data was carried out using TBSS version 1.2 [7]. A mean FA image was created and thinned to produce a mean FA skeleton representing the centers of all tracts common to the group. Aligned FA data from each subject were then projected onto this skeleton and the resulting data were fed into voxel-wise cross-subject statistics.

After achieving non-linear registration and stages of skeleton formation using FA images, projection vectors were estimated from each individual subject onto the mean FA skeleton. The non-linear warps and skeleton projection were then applied to the MD. The resulting statistical maps with a threshold at *p *< 0.05 were corrected at the cluster level for multiple comparisons.

After achieving non-linear registration and stages of skeleton formation using FA images, projection vectors were estimated from each individual subject onto the mean FA skeleton. The non-linear warps and skeleton projection were then applied to the MD, axial eigenvalue (i.e. λ1) and radial eigenvalue (i.e. (λ2 + λ3)/2) images separately. The resulting statistical maps with a threshold at *p *< 0.05 were corrected at the cluster level for multiple comparisons.

### SPECT methodology

The study subjects were injected with 740 MBq (20 mCi) of ^99 m^Tc-ethyl cysteinate dimer (ECD; Neurolite, Dupont, USA) in a quiet and dimly lit room. One hour after injection, brain SPECT images were acquired on a rotating 3-detector gamma camera (MULTISPECT, Siemens, Germany) with fan-beam collimators. The following acquisition parameters were used: 360° rotation, 90 projections at 128 × 128 matrix, 50-s acquisition time per projection. Filtered back projection with a Butterworth filter produced trans-axial sections, which were corrected for attenuation using Chang's attenuation correction method [12]. The sections were then re-oriented and made parallel to the orbito-meatal line. After realignment and co-registration with the normal template, a mean SPECT map was created for the five patients.

### Statistical analyses

Neuropsychological test scores were analyzed using non-parametric tests since the amount of data was small and not normally distributed. The primary measures of interest were the total scores of CASI as these were more stable than individual test scores.

Analyses carried out on the axial and radial eigenvalues were extracted on the aligned data based on the regions showing differences between patients and controls in FA values. Correlations between CASI total scores and FA were carried out using Spearman's rank order correlation. The statistical analysis was conducted using the Statistical Package for Social Sciences software package (version 13 for Windows^®^, SPSS Inc, Chicago, IL). A *p *value less than 0.05 was considered statistically significant.

## Results

### Cognitive tests and clinical features

The patients all scored below 5% in CASI total scores and sub-domains as compared to the controls (Table 1). All five patients received chenodeoxycholic acid (CDCA) treatment (750 mg/day) after the diagnosis of CTX was made.

### Conventional MRI findings

**Figure 1:**
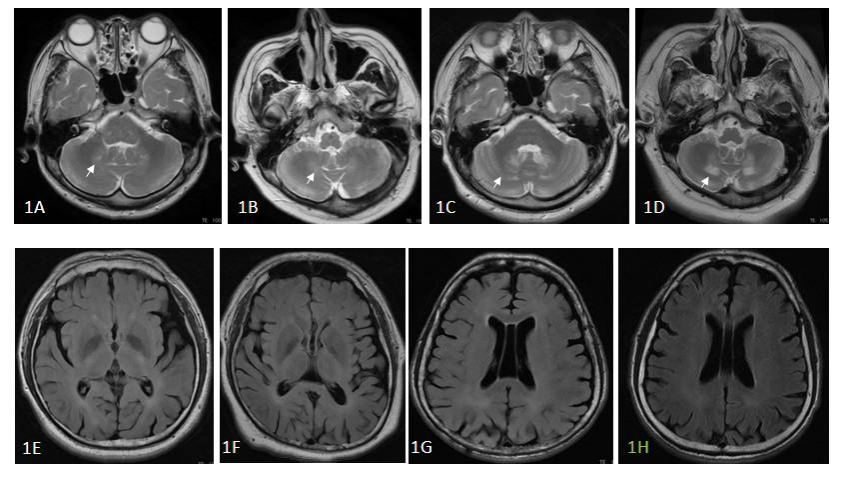
**Conventional magnetic resonance images (MRI) results**. MRI shows peri-dentate high signal intensities in the cerebellum in Cases 1 **(1A) **and 2 **(1C) **at diagnosis and their extension four years later (Cases 1 **(1B) **and 2 **(1D)**). Fluid attenuated inversion recovery images show equivocal high signal intensities in the peri-ventricular regions in Cases 1-4 **(1E-1H)**.

T2-weighted images showed characteristic dentate nuclei and changes in the surrounding WM signals in Cases 1 and 2 on first evaluation (Figures 1A and 1B). The WM signals had progressed 4 years later (Figures 1C and 1D). Equivocal high signal intensities were detected in the peri-ventricular region of the temporal lobes (Figures 1E and 1F representing Cases 1 and 2) and parietal-occipital sub-cortical regions (Figures 1G and 1H representing Cases 3 and 4) by FLAIR pulse sequences. Cases 2-4 had patent septum pellucidum with the presence of a 5th ventricle. Other imaging findings with longitudinal changes are listed in Table 1.

### Gray matter atrophy were parallel with ECD-SPECT results

**Figure 2:**
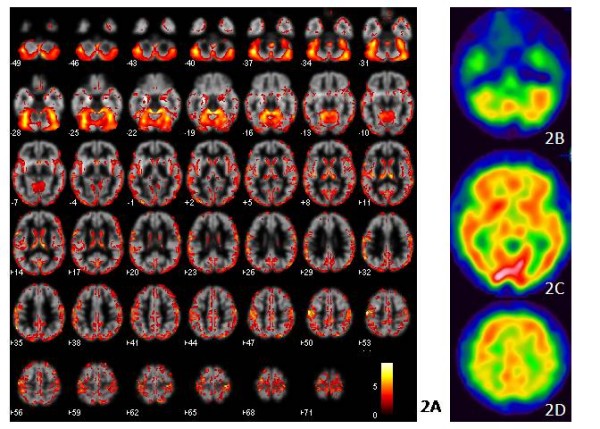
**Voxel-based morphometry analysis**. Maps of the t-value (whole brain voxel analysis at *p *< 0.05 FWE corrected for multiple comparisons) render on the gray map template showing diffuse cortical cerebellar gray matter volume loss **(2A) **and these atrophy patterns are correlated with single photon emission computed tomography results **(2B-2D)**.

There was an extensive and symmetrical GM loss in the cerebellar hemispheres and vermis of the patients as compared to the controls (p < 0.05 FWE corrected for multiple comparisons) (Figure 2A). There were additional clusters of decreased GM volume in the temporal, occipital, peri-Sylvian and basal ganglia regions. The atrophy patterns were parallel to the findings of hypo-perfusion shown in SPECT study (Figures 2B-2D).

### WM tracts

The anterior and posterior corpus callosum, superior and inferior longitudinal fasciculus, superior and inferior fronto-occipital fasciculus, uncinate fasciculus, dorso-lateral pre-frontal U fibers, internal capsule, fornix, medial lemniscus, superior, middle and inferior cerebellar peduncles, cortico-spinal tract, cerebral peduncles, optic radiation, and WM surrounding the dentate nucleus were the regions with reduced FA in the CTX group. Regions with MD increment were more extensive than FA and included the anterior and posterior corpus callosum, orbito-frontal and cortico-spinal tracts, internal capsule, external capsule, cerebellar pontine connection, midline cerebellum, thalamus and brain stem, and the cerebral peduncle.

**Figure 3:**
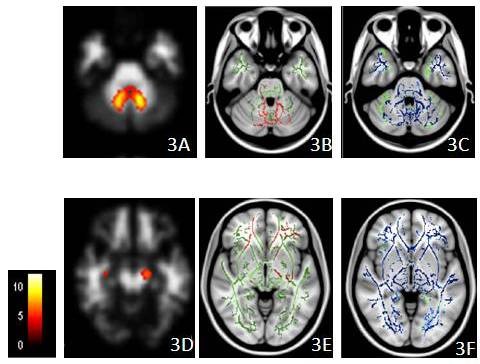
**Clusters with atrophy and white matter changes**. Clusters with atrophy by voxel-based morphometry (VBM) (**3A **and **3D**), reduced fractional anisotropy (**3B **and **3E **in red) and increased mean diffusivity (**3C **and **3F **in blue) in the patient group overlaid on the mean skeleton map (in green) (all *p *< 0.05, corrected at the cluster level). The colored bar indicates the t value in the VBM study. The peri-dentate region and left cerebral peduncle are the two regions that show overlapping across three measurements, while the MD changes are more extensive.

**Figure 4:**
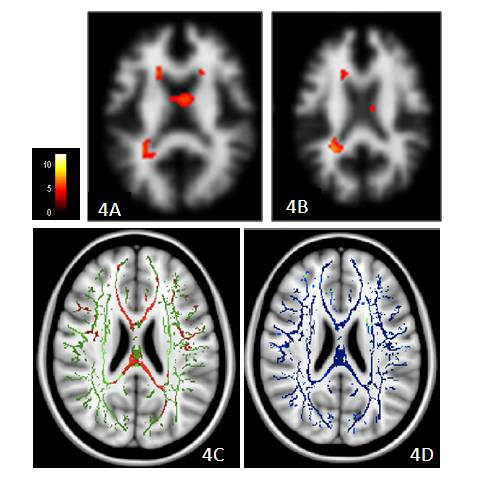
**Changes in fractional anisotropy (FA) and mean diffusivity (MD)**. Changes in FA and MD precede voxel-based morphometry (VBM) in the peri-ventricular white matter region. Clusters with atrophy by VBM (**4A **and **4B**), reduced FA (**4C **in red), and increased MD (**4 D **in blue) in the patient group overlay on the mean skeleton map (in green) (all *p *< 0.05, corrected at the cluster level). The colored bar indicates the t value in the VBM study.

The peri-dentate regions (Figures 3A-3C and left cerebral peduncle (Figures 3D-3F were the regions sharing overlap in WM VBM, FA and MD. WM atrophy was located in the frontal and occipital horn (Figures 4A and 4B), while FA and MD were affected in the anterior and posterior corpus callosum (Figures 4C and 4D).

**Figure 5:**
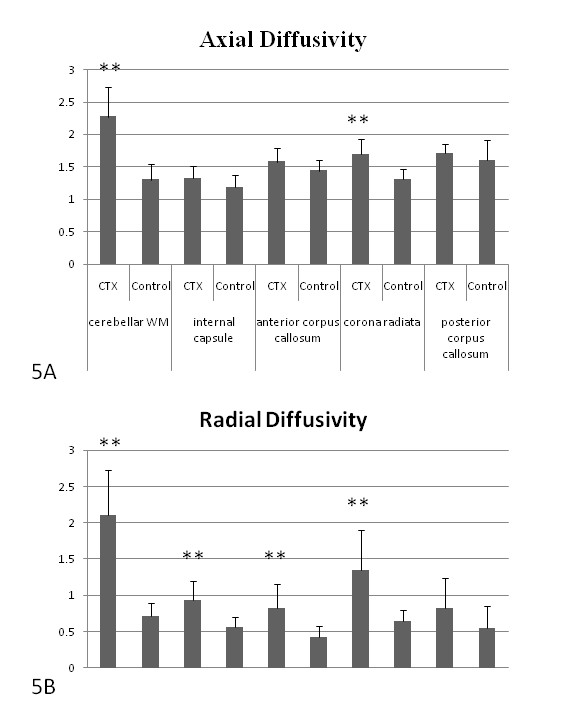
**Analysis of axial and radial eigenvalues in selected regions**. Regions with reduced fractional anisotropy (FA) are shown by increased axial and radial eigenvalues in the patient groups (**p < 0.001).

Increased axial and radial eigenvalues in the patient group contributed to regions with decreased FA (Figure 5). There were significantly increased axial eigenvalues in the peri-dentate and upper corona radiata regions as compared with controls (Figure 5A). There were also increased radial eigenvalues in the peri-dentate regions, internal capsules, anterior corpus callosum, and corona radiata (Figure 5B).

### Correlation of neuropsychological tests with FA

A correlation study between CASI scores with WM FA was conducted to examine the relationship between WM integrity and neuropsychological function. The rational why we selected CASI total score was because it represents a global assessment of cognitive performance. There were significant correlations between CASI and cerebellar WM (rho = 0.937, *p *= 0.0001), left posterior internal capsule (rho = 0.435, *p *= 0.017), left anterior internal capsule (rho = 0.496, *p *= 0.007), left anterior corpus callosum (rho = 0.613, *p *= 0.001), right anterior corpus callosum (rho = 0.420, *p *= 0.021), left upper corona radiata (rho = 0.514, *p *= 0.005), right upper corona radiata (rho = 0.469, *p *= 0.01), left posterior corona radiata (rho = 0.402, *p *= 0.026) and right posterior corona radiata (rho = 0.361, *p *= 0.041).

## Discussion

With the advent of molecular genetic analysis, the role of conventional MRI in diagnosing CTX has been considerably reduced [1]. Despite this, quantitative assessment of WM integrity is still important clinically since neuronal and mitochondrial dysfunction are known to occur in tissues that appear normal [15]. The cross modality comparison in this study indicates that CTX features in the brain are complex and structural imaging analysis such as diffusion measures and VBM may provide complimentary information to conventional MRI.

Combined with VBM and SPECT results, several patterns are observed in our study. First, the cerebellum, especially the peri-dentate region, is the major target of involvement in CTX both functionally and structurally. Second, despite the extensive GM involvement, hypo-perfusion in SPECT is consistent with the results of GM atrophy with a higher t value, which suggests that GM atrophy plays a key role in decreased perfusion. Third, decreased FA and increased MD in the left cerebral peduncle, bilateral peri-ventricular and peri-dentate regions correspond with the WM atrophy shown by VBM, suggesting irreversible processes in these selective regions. Changes in diffusion preceded VBM in the anterior/posterior corpus callosum and other WM regions suggesting improved sensitivity of DTI in detecting WM changes. Lastly, although treatment with CDCA is reported to delay or even reverse disease progression [16-18], progressive peri-dentate WM changes observed in our longitudinal study may argue otherwise.

The current study reports increased diffusivity in normal appearing WM, beyond the affected WM regions where both decreased FA and increased MD were noticeable. In theory, changes in diffusion can be attributed to an increase in extra-cellular space, more myelin permeability with axonopathy or the presence of elements other than myelin sheaths within these structures, which have a greater diffusion capacity than water [19,20]. The VBM results here show regions of WM atrophy that may explain the effect of tissue atrophy to increase extra-cellular space and change water diffusion. In most case reports on CTX, WM lesions are referred to as demyelinating [21-24]. However, this term is considered relatively loose because there is also extensive axonal loss in the cerebellar and midbrain lesions. Pathology reports by both Soffer *et al *[25] and Pop *et al *[26] delicately describe the pathology of WM tract and emphasize the examination of myelin and axon integrity. Their reports suggested that demyelination did not result from abnormal myelin composition in CTX. Instead, myelin loss is secondary to axonal loss. They also indicated that the occurrence of axonal spheroids in the brain stem suggested the pathology of WM as axonal degeneration.

The role played by axonal injury may also partly explain the irreversibility and progression of cognitive decline in the study patients despite CDCA treatment. Extensive astrocytosis accompanied by sheets of macrophages and crystalline clefts, that are extensively reported in the literature on CTX pathology, may cause a change in water diffusion [21-24]. However, based on the fact that the diffusion coefficients of these elements are smaller than water, FA and MD changes in our study were less likely to be from the contribution of these substances. Combining the pathology features previously reported, demyelination, axonopathy, and tissue atrophy all occur in CTX.

Because diffusion indices such as FA and MD are derivatives of three tensor eigenvalues, the correct interpretation of diffusion changes should consider the contribution of each eigenvalue. The study results show that increased MD from DTI data are contributions by the increments of axial and radial eigenvalues. Decreased FA can be attributed to the fact that the increment in radial diffusivity is larger than the corresponding ones. Increased radial diffusivity is likely to reflect myelin damage [19]. Increased axial diffusivity is related to axonopathy, which has previously been observed in neurologic disorders with axonal lesions [20,21]. Therefore, analysis of axial and radial diffusivity also suggests the presence of both demyelinating and with axonopathic lesions in CTX.

Only one case report [27] in the literature mentions SPECT findings in CTX, which showed cerebellar and bi-parietal hypo-perfusion. The current study results not only reiterate previous findings but also show that the perfusion patterns reflected the pattern of GM atrophy. The radioactivity of ECD SEPCT is dependent on regional blood perfusion [28] and is reasonable to show the relationship with the atrophic pattern. However, from CTX pathology literature, the cyto-architecture of the cerebral cortex is preserved, showing no apparent neuronal loss or any significant neuronal changes [15]. It is presumed that the "dying back" phenomenon is a mechanism of GM atrophy because these neurons are unable to maintain metabolic integrity. The major involvement of the pyramidal tract in our FA results with greater peri-Sylvian atrophy in VBM support this notion. The toxic accumulation of cholestanol, crystalline clefts, and the presence of gliosis and macrophages may cause axonal damage and further neuronal degeneration. The greater atrophy of WM in the posterior peri-ventricular region is also evidence of this hypothesis.

There is increasing evidence that WM abnormalities are associated with dementia [29]. Our study reveals that in addition to cortical atrophy, there is also damage in the major associative fiber tracts in CTX. These tracts are unique to humans and cannot be identified as discrete fiber bundles in rodents, primates, or human fetal brains [30]. The net effect of major associative fiber injury in CTX suggests the impairment of higher cognitive functions, which are also well demonstrated in the current correlation results. As regards the neuropsychological presentation, the five patients already had mild to moderate mental retardation upon diagnosis. Although the imaging resolution of this DTI study does not reveal cellular-level connectivity information for the brain cortex, it is still of great interest to investigate deficits associated with these bundles. Therefore, in addition to GM involvement, the cognitive presentations with the correlation study of the CTX patients can be partly explained by the poor integrity of these fiber tracts. The involvement of the associative tracts supports the theory that CTX is a developmental disorder [30].

There are several limitations to this study. First, this study enrolled a small number of patients. Because of the rarity of this disorder, the interpretation presented here might represent the results related to the two families. Whether it can be applied to the general population of CTX families requires a larger population study. Second, we cannot provide histology proof in this study because this is a cross sectional study on human subjects. Therefore, the interpretation of the pathological changes through neuroimaging findings (as reflected in changes in axial and radial eigenvalues) cannot be proved. Nonetheless, the results are consistent with previous pathology studies where myelin damage is constant with or without axonal destruction. It is worth pointing out that a direct correlation between axial and radial diffusivity parameters and the microscopic pathology of white matter injury has not been conclusive. As such, interpretation of changes in axial/radial eigenvalues as demyelination or axonopathy [31,32] should be treated with great care, especially in regions where neuron fibers cross [33].

## Conclusion

This study shows that DTI and VBM can provide complementary information regarding the GM or WM involvement in CTX, and that they are more sensitive than conventional neuroimaging studies. From imaging study results, WM involvement including demyelination, axonal changes and tissue loss suggest irreversibility, especially in the peridentate areas. Major associative fiber involvement as well as cortical atrophy account for the broad spectrum of cognitive deficits in these patients. The cognitive decline and image changes in conventional MRI indicate neuro-toxicity and possible irreversibility of this disease despite using CDCA.

## Competing interests

The authors declare that they have no competing interests.

## Authors' contributions

CCC designed the study, carried out the statistical analysis of the images, and drafted the manuscript. CCL and CFC carried out the DTI acquisition and interpretation of the DTI data. JJW carried out the statistical analysis of DTI imaging. SHH carried out the SPECT acquisition and statistical analysis of SPECT imaging. CHL, MCT and CWH carried out clinical data evaluation and statistical analysis. CFC obtained the neuropsychological data and carried out statistical analysis. WNC conceived the study design and critically reviewed the manuscript. All authors have read and approved the final manuscript.

## Pre-publication history

The pre-publication history for this paper can be accessed here:

http://www.biomedcentral.com/1471-2377/10/59/prepub
